# Pelvic Surgery

**DOI:** 10.1155/2012/287096

**Published:** 2012-09-18

**Authors:** Constantine P. Karakousis, Harold Wanebo

**Affiliations:** ^1^Department of Surgery Buffalo General Hospital, 100 High Street, Buffalo, NY 14203, USA; ^2^State University of New York at Buffalo, 408 Capen Hall, Buffalo, NY 14260, USA; ^3^Landmark Medical Center, Woonsockett, RI, USA; ^4^Boston University, One Silber Way, Boston, MA 02215, USA

The present issue of the International Journal of Surgical Oncology on Pelvic Surgery contains a series of articles on prostate cancer, gynecologic malignancies, and rectal cancer.

The article on “radical prostatectomy as a first-line treatment in patients with initial PSA >20 ng/mL” by Hinev et al. reports on patients diagnosed with prostate cancer (PCa) and PSA >20 ng/mL. The elevated PSA level is considered an adverse prognostic factor in PCa often regarded as contraindication to radical surgery. The authors purported to estimate the impact of radical prostatectomy (RP) on biochemical-recurrence-(BCR-) free and cancer specific survival (CSS) for these patients. Men in this group had significantly lower 10-year BCR-free and CSS rates than patients with initial PSA <20 ng/mL (20.7% versus 79.6%/*P* < 0.001/and 65% versus 87.9%/*P* = 0.01, resp.). Pathological stages were found to be independent predictors of PSA failure in men with PSA >20 ng/mL. Patients with favorable prognostic variables (pT2, NO) had significantly longer disease-free and overall survival similar to those with initial PSA <20 ng/mL. High PSA values do not indicate poor prognosis uniformly and therefore along with patients with organ-confined PCa and negative lymph nodes may benefit from RP. In one series more than 50% of patients with initial PSA values above 20 ng/mL had undetectable PSA values over the first 5-years after RP. Similar results have been reported in other series with RP used as monotherapy. Neoadjuvant hormonal therapy is no longer recommended for patients subjected to radical surgical treatment. The authors suggest further studies in patients with initial PSA values >20 ng/mL and use of RP in order to verify the results of their study. 

The article “Total pelvic exenteration (PE) for gynecological malignancies” by Diver et al. describes PE as the en-bloc resection of pelvic organs including reproductive structures, bladder, and rectosigmoid. It is commonly indicated for advanced primary or locally recurrent cancer without evidence of metastatic disease or elements which preclude resection. Major complications occur in as many as 50% of the patients. In carefully selected patients with gynecologic cancer PE can be curative. Separate stomata for urine and fecal diversion and the use of omentum to protect and cover the denuded surfaces and more recently development of techniques to remove involved pelvic side wall have increased the chance of curative surgery. Laparoscopic and robotic-assisted technology has improved operative recovery while a 5-year survival rate of about 50% has been reported. Various techniques for functional neovaginas have been developed. Anterior and posterior exenteration techniques are described. PE is usually performed with curative intent but palliative PE has been used in cases mainly of severe radiation necrosis. The authors describe extensively complications and quality of life after PE and provide useful overall information in doing PE for gynecological malignancies. 

The article by A. F. R. Cubal et al. on “Fertility-sparing surgery for early-stage cervical cancer” reviews data on procedures for fertility preservation, that is, vaginal and abdominal trachelectomy. The overall oncologic safety is good compared to radical hysterectomy offered traditionally and the obstetrical outcomes are promising. Good selection of patients and complete information with a detailed informed consent is required. The authors describe the eligibility criteria in terms of tumor dimensions, depth of invasion, type and grade and lymphovascular space involvement. The procedures of vaginal and abdominal radical trachelectomy are described, as well as the follow-up and use of less radical procedures. Neoadjuvant chemotherapy has been employed in women with larger cervical lesions (>2 cm) in order to decrease the tumor size and provide a more conservative endocervical tissue resection. In conclusion, radical vaginal trachelectomy is a well-established safe procedure for early cervical cancer (<2 cm) with good oncological and obstetrical outcomes and low morbidity-mortality rates. Open abdominal or laparoscopic approaches are increasingly used which along with robotic surgery will provide more surgical options for these patients. 

The article on “The Retrograde and Retroperitoneal Totally Laparoscopic Hysterectomy for Endometrial Cancer” by E. Volpe et al. describes their experience for total laparoscopic hysterectomy based on completely retrograde and retroperitoneal technique for surgical staging and treatment of endometrial cancer. The technique used was based on a combination of a retroperitoneal approach with a retrograde and lateral dissection of the bladder and retrograde culdotomy with variable resection of parametrium. The authors' laparoscopic technique and retroperitoneal approach allows control of the main uterine vessels, constant monitoring of the ureters and exposure and removal of the lymph nodes as needed. The procedure has been used in 95 patients (Jan 2002–Dec 2011). It has cost savings implications and does not require a uterine manipulator which is, when used, a concern for possible dissemination of tumor. 

The article on “Intersphincteric resection and coloanal anastomosis in the treatment of distal rectal cancer” by Gokhan Cipe et al describes clearly the technique of intersphincteric resection providing sphincter saving surgery for patients with distal rectal cancer as an alternative to abdominoperineal resection (APR). The extent of the intersphincteric resection (ISR) is distinguished into partial, subtotal and total. When the tumor spread is to or beyond the dentate line, total ISR should be done. If the distal edge of the tumor is more than 2 cm from the dentate line, subtotal ISR is performed, the distal resection margin being between the dentate line and the intersphincteric groove. When there is sufficient distal surgical margin, the distal line of resection can be on or above the dentate line (partial ISR). The common complications of ISR are anastomotic leakage, stricture, fistula, pelvic sepsis, bleeding etc. ISR has rates of local recurrence between 2% and 3%. The 5-year survival with ISR has been reported to be about 80% and disease-free survival 69%. In some studies the survival after abdominoperitoneal resection (APR) was lower than after ISR. Complete continence after ISR is observed in 30% to 86%, while fecal soiling occurs in 15% to 63% of patients. The authors conclude that sphincter-saving surgery may be the treatment of choice for distal rectal cancer which is of early stage, well differentiated or underwent objective regression after neoadjuvant therapy.

 The article on “The role of secondary surgery in recurrent ovarian cancer” by D. Lorusso et al. reports that although primary complete cytoreduction and adjuvant Platinum-Paclitaxel chemotherapy is a well established treatment for intraperitoneal spread of ovarian cancer, the 5-year survival being about 30% the role of secondary cytoreductive surgery for recurrent disease is controversial. The authors discuss on how to identify patients most likely to benefit from a secondary cytoreduction and the prognostic factors for survival of whom complete debulking is the strongest predictor. Absence of ascites and reintroduction of platinum are also associated with prolonged survival. In addition, the authors address the issue of cytoreductive surgery and hyperthermic intraperitoneal chemotherapy (HIPEC). HIPEC has attracted considerable interest due to promising results in peritoneal colon cancer carcinomatosis but in ovarian carcinomatosis the survival benefit is not evident requiring a well designed prospective randomized phase III Trial. The authors believe that there is a role for secondary cytoreductive surgery in well selected patients (absence of ascites, good performance status and complete debulking). 

*Constantine P. Karakousis* *Constantine P. Karakousis* *Harold Wanebo* *Harold Wanebo*

